# Costs related to cardiac arrest management: a systematic review protocol

**DOI:** 10.1186/s13643-017-0599-z

**Published:** 2017-10-17

**Authors:** Guillaume Geri, Joshua Gilgan, Carolyn Ziegler, Wanrudee Isaranuwatchai, Laurie J. Morrison

**Affiliations:** 1 Rescu, Li Ka Shing Knowledge Institute, St Michael’s Hospital, 30, Bond Street, Toronto, ON M5B 1M8 Canada; 2 University of Toronto, Toronto, ON Canada; 3 Health Sciences Library, St Michael’s Hospital, Toronto, ON Canada; 4 Centre for Excellence in Economic Analysis Research (CLEAR), St. Michael’s Hospital, Toronto, ON Canada; 5 Institute of Health Policy, Management and Evaluation, University of Toronto, Toronto, ON Canada

**Keywords:** Out-of-hospital cardiac arrest, In-hospital cardiac arrest, Costs, Inpatient costs, Pre-hospital costs

## Abstract

**Background:**

Each year, about 500,000 people suffer a cardiac arrest (either out-of-hospital or in-hospital) in the USA. Although significant improvements in survival have occurred through the implementation of complex high-quality protocols of care, global costs related to such management are not clearly described.

**Methods:**

We will undertake a systematic review of the published literature on costs related to the acute phase of cardiac arrest management (from collapse to hospital discharge). The search will cover the period 1991 to present, and we will include studies written in English or in French involving patients with cardiac arrest of all ages, settings (in- and out-of-hospital arrest), countries, and etiology (including traumatic). The primary outcome will include estimates of costs related to cardiac arrest patients’ management in various categories (e.g., resuscitation process, in-hospital management as well as rehabilitation and long-term care facilities) and perspectives (e.g., hospital, societal, or third-payer perspective). Study selection will follow the Preferred Reporting Items for Systematic Reviews and Meta-Analyses (PRISMA) guidelines, and data quality will be assessed by questions adapted from the Drummond economic evaluation checklist.

**Discussion:**

This review will provide an estimate of costs related to cardiac arrest management according to the different components of such a management as well as total costs.

**Systematic review registration:**

International Prospective Register of Systematic Reviews PROSPERO CRD42016046993

## Background

Each year, about 320,000 adult people in the USA will suffer an out-of-hospital cardiac arrest [1]. In the USA and other developed nations, there is a wide variation in the approach to the treatment of cardiac arrest patients. For example, in prehospital care, the Anglo-American system (in countries such as the USA, Canada, or Australia) “brings the patient to the hospital,” whereas the French-German system “brings the hospital to the patient” [2–4]. Once admitted, implementation of multifaceted interventions such as targeted temperature management (TTM) or immediate coronary angiography with percutaneous coronary intervention in successfully resuscitated cardiac arrest patients has led to greatly improved health outcomes of such patients across countries [5–8]. Although the increasing complexity of care is widely believed to have enabled significant improvement in the survival of cardiac arrest patients, it has come at a significant financial cost as well, which limits a widespread adoption and results in variability in care.

Although there is some literature demonstrating the costs of individual components of cardiac arrest care, such as TTM [9], resuscitation-specific costs [10, 11], or specific subgroups of care [12], a comprehensive review of all the costs associated with treating cardiac arrest does not exist. This may be in part due to the complex, multidisciplinary care required by these patients, which means that costs must be collected from many different sources. There is also a wide variation in the costing methodology of such studies, leading to difficulties in comparing or combining cost estimates.

This review will therefore seek to provide a comprehensive systematic review of the literature related to determining the costs of managing cardiac arrest, including the costs from prehospital, in-hospital, rehabilitation, and long-term care perspectives.

## Objective

We aim to conduct a systematic review, reporting the costs of treating patients with cardiac arrest. This review will focus on costs of the acute phase of cardiac arrest management (including prehospital, in-hospital part, and rehabilitation) from different perspectives (e.g., hospital, societal, or third payer perspective).

## Methods

We will undertake a systematic review of the published peer-reviewed journal articles.

### PICO question

The PICO question is the following:

- *P*: cardiac arrest patients (i.e., in-hospital and out-of-hospital cardiac arrest, both adult and pediatric). We will distinguish adults from senior (i.e., older than 65 years old) in regard to the specific particularities of such a subgroup of patients.
- *I*: any intervention to do with cardiac arrest will be taken into consideration in this systematic review: public access defibrillation, bystander cardiopulmonary resuscitation (CPR), dispatcher assisted CPR, targeted temperature management and coronary angiography (and percutaneous coronary intervention), and post-cardiac arrest bundles of care (i.e., rehabilitation and long-term care facilities).
- *C*: patients who do not have the intervention
- *O*: cost estimates

### Study selection

We will include studies meeting any of the following inclusion criteria: patients with a cardiac arrest regardless of age, setting (in- and out-of-hospital cardiac arrest), countries, and any etiology (including traumatic) and costs clearly indicated as a currency value and year. If year is not explicit, we will assume that the year of publication could be used to adjust for inflation rate.

Both full and partial economic evaluation will be considered for inclusion in the present review, except those relying on modeling analyses.

We will exclude studies with any studies meeting any of the following inclusion criteria: studies examining sudden infant death syndrome or stillborn infants, modeling studies without primary collected cost data, all reviews without original data, conference abstracts, and syllabi.

### Outcome measures

The primary outcome will include estimates of the range of total absolute costs related to care of cardiac arrest patients’ reported at an individual level.

The secondary outcomes will include description of costs related to the different time intervals of setting of care (i.e., prehospital, in-hospital, rehabilitation, long-term care facility, and outpatient) over which the economic evaluations have been undertaken.

### Search strategy

With the help of an information specialist, we will design our search strategy to capture all potential costs associated with care of the cardiac arrest patient. Our search strategy will include (but will not be limited to) variations on the terms “cardiac arrest,” “cardiopulmonary resuscitation,” and “ventricular fibrillation” as well as validated search filter [13, 14] for economic-related terms including “cost,” “economics,” “expenditure,” and “value” amongst others. Our search will be limited to original published manuscripts in peer-reviewed journals, dating back to 1991, when the first American Heart Association guidelines for cardiac arrest management were published. We will include only human studies. Our search will be carried out in MEDLINE, Embase, Ovid EBM reviews (ACP Journal Club, Cochrane Database of Systematic Reviews, Database of Abstracts of Reviews of Effects, Cochrane Central Register of Controlled Trials, Cochrane Methodology Register, Health Technology Assessment, NHS Economic Evaluation Database), and Web of Science (Science Citation Index Expanded). Specific search strategies will be employed for each database.

### Study selection

After retrieving initial results, duplicates will be removed and the remaining studies will be loaded into the online systematic review software Covidence [15]. Two authors (GG and JG) will independently perform the title and abstract screening. Conflicts will be tracked to determine the rate of agreement. Conflicts will be resolved by a consensus discussion between the two reviewing authors, airing on the side of inclusivity. A third author (LJM) will be consulted if consensus cannot be reached. Full texts will then be retrieved for further review of inclusion based upon above criteria. We will screen the references of included studies as well as excluded modeling studies for potential additional publications.

### Critical appraisal of methodological quality and data extraction

We will evaluate the risk of bias using the Drummond checklist, as recommended by the Cochrane Handbook Chapter 15 [16]. Briefly, the Drummond checklist explores the different domains of cost analysis, i.e., study design, data collection and analysis, and interpretation of results [17].

Data will be extracted onto a customized data extraction sheet by two independent reviewers (JG and GG). Any disagreement will be resolved through discussion, with arbitration by a third reviewer (LJM) if necessary.

Variables to be extracted will include:

- First author, year of publication, and journal
- Country of origin
- Setting for care delivery (prehospital, inpatient, outpatient, long-term care, and rehab)
- Baseline cardiac arrest characteristics (age, gender, initial shockable rhythm, location of cardiac arrest, presence of witness, bystander CPR, collapse to discharge survival rate) and outcomes of cardiac arrest (admission to discharge survival rate, return of spontaneous circulation (ROSC)). Initial shockable rhythm, presence of witness, and bystander CPR are binary variables and will be presented as the proportion of patients who met this condition during the acute episode.
- Setting of cardiac arrest (out-of-hospital or in-hospital) and cause of cardiac arrest (medical or traumatic)
- Age category of patients (pediatrics, adults, and adults older than 65 years old)
  - Re-arrest in the hospital prevalence

The different settings will be defined as follows: the prehospital setting from the emergency service call until the arrival to the emergency department, the in-hospital setting including stays at both emergency department and inpatients wards, and the post-discharge setting including outpatients, rehabilitation, and long-term care facilities. A breakdown by country will be performed as well.

Authors of retrieved studies will be contacted if data extraction resulted in missing values.

Cost data will be abstracted as follows:

- Reported currency, reported or presumed currency year
- Perspective of the analysis (i.e., payer): public payer, hospital, third party, or societal
- Costs for each setting for care delivery (could include more than one in each study) the authors assessed, as well as total costs
- For inpatient costs, we will also collect the main components of costs that have been taken into account, when indicated, e.g., rooming charges, equipment, medications/pharmacy, laboratory, and physicians’ fees including overhead cost and their definition which may differ across studies
- For in-hospital cardiac arrest, costs related to the resuscitation process will be collected separately when available

Costs will be extracted in “local currency.” After extraction, to make findings comparable, costing data not in US dollars will be converted to equivalent using the Organization for Economic Co-operation and Development “Purchasing Power Parities (PPP) for gross domestic product” dataset for the year of publication (*http://www.oecd.org* */*). When a PPP from OECD is not available, conversion rates given within the manuscript details will be used. The healthcare-specific Consumer Price Index (*http://www.bls.gov/cpi/*) will then be used to adjust for inflation. All values in the review will therefore be presented in 2015 US dollars. Costs related to the management of these different settings will be reported as they are provided in the manuscript. Overall costs will be reported as well if they are provided in the original paper but will not result of the sum of the different components described above.

Findings will be presented in tables according to the study design perspective, the survival status at hospital discharge, the setting of arrest (out-of-hospital or in-hospital), the population (adults/pediatrics and adults older than 65 years old), and the time frame of cardiac arrest management (prehospital, in-hospital, rehabilitation, long-term care facilities, and outpatient). A breakdown by type of health care system (i.e., Anglo-American vs. French-German system) will be performed as well.

### Data sharing agreement

This systematic review protocol is registered with the PROSPERO International Prospective Register of Systematic Reviews (http://www.crd.york.ac.uk/prospero) as CRD42016046993 and will be reported using Preferred Reporting Items of Systematic Reviews and Meta-Analyses (PRISMA) guidelines in the peer-reviewed literature. [18]

## Discussion

Cardiac arrest is a major public health issue, and costs related to its management have not been extensively described. Most of the previously reported works provided incomplete information, either by providing costs related to a specific component of cardiac arrest management or by focusing on a procedure. This review may provide interesting medico-economic considerations about out-of-hospital as well as in-hospital cardiac arrest management. This will also provide contrasted estimates of costs related to the management of adults and pediatric patients who suffered a cardiac arrest. Last, this may provide the very first overview in the field.

## Abbreviations

CPR: Cardiopulmonary resuscitation
PPP: Purchasing power parities
ROSC: Return of spontaneous circulation
TTM: Targeted temperature management
